# Task-Oriented Inference Framework for Lightweight and Energy-Efficient Object Localization in Electrical Impedance Tomography

**DOI:** 10.3390/s26082570

**Published:** 2026-04-21

**Authors:** Takashi Ikuno, Reiji Kaneko

**Affiliations:** Department of Applied Electronics, Graduate School of Advanced Engineering, Tokyo University of Science, Tokyo 125-8585, Japan

**Keywords:** electrical impedance tomography, object localization, task-oriented inference, edge computing, energy efficiency

## Abstract

Electrical Impedance Tomography (EIT) is a promising non-invasive sensing technique, yet its practical application in resource-constrained environments is often limited by the high computational cost of inverse image reconstruction. To address this challenge, we focus on specific sensing objectives rather than full image recovery. In this study, we propose a lightweight, task-oriented inference framework for object localization in EIT that bypasses the need to solve computationally expensive inverse reconstruction problems. This approach addresses the high computational demands and hardware complexity of conventional iterative methods, which often hinder real-time monitoring in resource-constrained edge computing environments. Training datasets were generated via finite element method (FEM) simulations for Opposite and Adjacent current injection configurations. A feedforward neural network was developed to independently estimate the radial and angular object positions as probability distributions. Our systematic evaluation revealed that the localization performance depends on the injection configuration and model depth; notably, the Opposite method achieved perfect classification accuracy (1.00) for radial estimation with an optimized architecture of four hidden layers, whereas the Adjacent method exhibited higher ambiguity. Results quantitatively evaluated using the Wasserstein distance show that the Opposite configuration produces more localized, unimodal probability distributions than the Adjacent configuration by utilizing current fields that traverse the entire domain. Compared with existing image-based reconstruction methods, including the conventional electrical impedance tomography and diffuse optical tomography reconstruction software (EIDORS ver.3.12), the proposed framework reduced energy consumption from 3.09 to 0.96 Wh, demonstrating an approximately 70% improvement in energy efficiency while maintaining a high localization accuracy without the need for iterative Jacobian updates. This task-oriented framework enables reliable, high-speed, and energy-efficient localization, making it well-suited for low-power EIT applications in mobile and embedded sensor systems.

## 1. Introduction

Electrical impedance tomography (EIT) has been widely studied as a non-invasive imaging technique, where the primary objective is to reconstruct spatial conductivity distributions by solving ill-posed inverse problems [1]. Owing to its safety and versatility, EIT has gained significant attention across diverse fields, ranging from medical diagnostics, such as pulmonary and breast imaging [2,3], to industrial process monitoring [4,5] and environmental sensing [6,7,8]. In recent years, most research in this field has focused on enhancing image resolution and reconstructive fidelity. Specifically, advanced methodologies such as convolutional neural networks (CNNs) [9] and generative adversarial networks (GANs) [10] have been introduced to overcome the inherent ill-posedness of EIT, achieving high-definition imaging that rivals traditional iterative solvers [1,11]. While these data-driven approaches have demonstrated superior performance in capturing complex spatial conductivity distributions, they typically require high-dimensional output spaces and significant memory overhead [11]. As highlighted in recent comprehensive reviews, such computational intensity poses a critical bottleneck for the novel frontier of wearables, where real-time monitoring must be balanced with strict energy budgets [11].

In many practical scenarios, however, EIT is required to operate under strict constraints on computational resources and power consumption, particularly in mobile, portable, or battery-powered sensing systems [11,12,13]. In these contexts, the computational burden of inverse-problem-based image reconstruction is a critical limitation. This is primarily due to the high energy cost of the iterative solvers required for full conductivity mapping, which hinders the deployment of autonomous edge-sensing devices.

Considering practical applications, however, high-definition shapes of internal objects are often unnecessary; instead, knowing the approximate location of an object is sufficient to achieve the sensing objective. Examples include bedside healthcare monitoring [14,15], infrastructure inspection [16,17,18], and distributed environmental sensors [19]. In these contexts, object localization is the most fundamental and essential task. However, conventional reconstruction-based approaches rely on iterative solvers, leading to substantial energy consumption even for such limited information [20,21,22]. Furthermore, while recent data-driven methods have attempted to directly regress object coordinates, they often fail to represent the spatial uncertainty or confidence of the estimation. Therefore, developing a task-oriented EIT method, specifically designed for localization with low power consumption and high processing speed, is of great importance for practical deployment. Table 1 summarizes the methodologies, primary outputs, and computational costs of existing representative EIT approaches alongside the proposed framework.

To address these challenges, we developed a task-oriented inference framework tailored for efficient object localization. As previously mentioned, while numerous studies have reported on high-resolution imaging [1], to the best of our knowledge, there have been no reports on methods specifically specialized for identifying only the location of objects within a domain. The transition to this task-oriented inference necessitates a strategic selection of the current excitation strategy, as the spatial distribution of the sensing field is primarily determined by the electrode configuration used for current injection. For a lightweight neural network to accurately extract features from a lower-dimensional input space, the object’s presence must induce a sufficiently detectable and uniform signal response across the entire domain.

The core of our proposed framework is a machine learning-based model designed to output object positions as independent probabilistic distributions over a partitioned polar-coordinate mesh (*r*, *θ*). This model was trained using a dataset of boundary voltage differences and ground-truth positions obtained via finite element method simulations. To optimize the sensing performance, we comparatively investigated the differences in probability distributions under two representative excitation strategies: the Adjacent method and the Opposite method. By shifting the objective from redundant image reconstruction to targeted task inference, we prioritize computational efficiency and energy savings. Consequently, the performance of our framework is evaluated through metrics critical for practical edge implementation, such as energy consumption and computational cost, rather than a direct numerical comparison of reconstruction accuracy with high-performance offline algorithms. Finally, we validated the effectiveness of our approach through experiments using a physical tank model with an object placed in an electrolyte, comparing our results with conventional inverse-problem-based reconstruction methods.

## 2. Materials and Methods

This section describes the design and implementation of the proposed task-oriented inference framework for EIT-based object localization. To achieve a lightweight and energy-efficient system, we shift the objective from full-scale image reconstruction to a direct spatial probability estimation. The methodology is structured into three primary phases. First, we define the forward problem and data generation process using finite element method (FEM) simulations to construct a robust dataset of boundary voltage differences. Second, we present the architecture and formulation of a feedforward neural network designed to map these voltage patterns directly to object existence probabilities over a partitioned mesh. Finally, we describe the experimental setup using a physical tank model and the metrics used to evaluate localization performance and energy efficiency. By integrating physical excitation strategies with a simplified inference model, this framework aims to bypass the computational overhead inherent in conventional inverse solvers.

### 2.1. FEM-Based Forward Simulation and Dataset Generation

**Figure 1:**
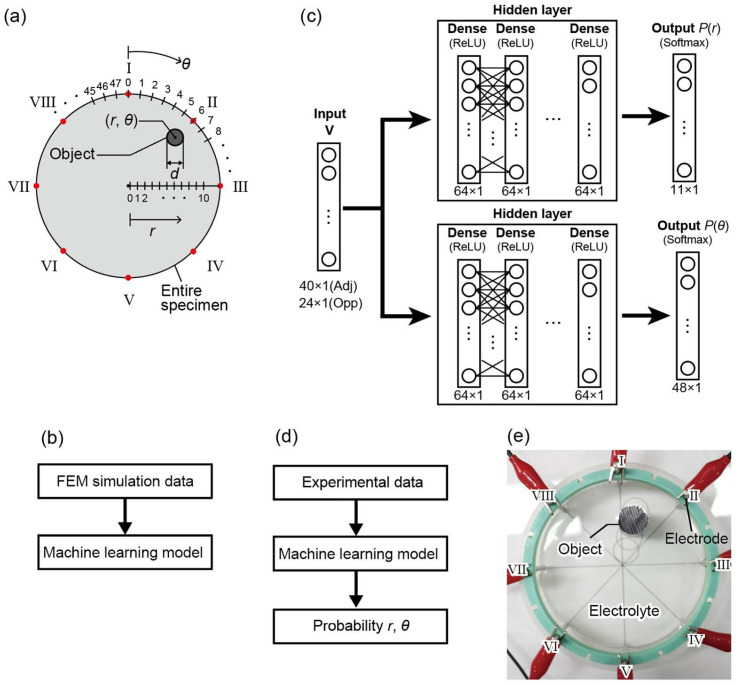
(**a**) Definition of polar coordinates (*r*, *θ*) and electrode numbering (*L* = 8). (**b**) Flowchart of the training dataset generation via FEM simulation. (**c**) Neural network architecture for *r*-estimation and *θ*-estimation. The number of hidden layers *N* was evaluated from 1 to 6. (**d**) Workflow of the implementation process for localization. (**e**) Experimental setup with a cylindrical container and electrodes.

In this study, we aim to develop a lightweight machine learning model that can estimate object locations with low computational cost without performing inverse-problem-based image reconstruction. To systematically generate training datasets for this purpose, numerical simulations based on the finite element method (FEM) were conducted for two current injection and voltage measurement configurations in EIT measurements, namely the Opposite and Adjacent methods [19]. Figure 1b shows the workflow for generating these training datasets.

The forward problem in EIT involves calculating the internal potential distribution *ϕ* when the conductivity distribution *σ* and the injected current *I* are known. This is governed by the following Laplace equation derived from Ohm’s law and the continuity of the current:

(1) $$\nabla \cdot \left(\sigma \nabla \phi \right)=0\;\;\;\;in\;\Omega$$

where Ω denotes the two-dimensional circular domain. To obtain boundary voltages, we consider the electrodes ϵ*_l_* (*l* = 1, …, *L*, where *L* = 8) placed on the boundary. The relationship between the electrode potential *V_l_* and the injected current *I_l_* is defined by the following boundary conditions:

(2) $$\int_{\epsilon_{l}}\sigma \frac{\partial \phi }{\partial n}dS=I_{l}$$

where *n* is the outward unit normal vector. While the complete electrode model is often used [23] to account for contact impedance, in this study, contact impedance was neglected to focus on the fundamental response of the task-oriented model.

Numerical simulations were performed using COMSOL Multiphysics 6.2 with a two-dimensional circular domain of 100 mm in diameter. Eight electrodes were equally spaced along the boundary, as shown in Figure 1a. Direct current (DC) was injected with a current density of 100 A/m^2^. The mesh consisted of free triangular elements and was locally refined near the electrodes and the object to ensure numerical accuracy. The background conductivity was set to 0.1 S/m, and a single circular object with a conductivity of 10^7^ S/m was embedded in the domain. The object diameter was varied from 1 to 49 mm.

The object location was defined in polar coordinates (*r*, *θ*), where *r* was discretized into 11 levels from 1 to 41 mm and *θ* ranged from 0° to 352.5° in 7.5° increments (see Figure 1a). These discretized values of (*r*, *θ*) were used as class labels in the machine learning model. For each object position, the boundary voltage differences *v_m_* (*m* = 1, …, *M*) were collected to form the input vector **V** = [*v*_1_, *v*_2_, …, *v_M_*]*^T^*.

In both the Opposite and Adjacent methods, the measured voltage difference between the *i*-th and *j*-th electrodes is defined as:

(3) $$v_{i,j}=\phi \left(\epsilon_{i}\right)-\phi \left(\epsilon_{j}\right)$$

In the Opposite method, current is injected between diametrically opposite electrodes, and the voltage differences are measured across three electrode pairs oriented perpendicularly to the injection axis. This configuration results in *M* = 24 measurements across eight sequential injection patterns. In contrast, the Adjacent method involves current injection through neighboring electrodes, with voltage differences measured across five adjacent electrode pairs, excluding the injection pair. In this case, the indices of the measured electrode pairs (*i*, *j*) follow the relationship *j* = (*i* mod 8) + 1 to ensure cyclic continuity among the eight electrodes. This Adjacent configuration yields a larger input vector with *M* = 40 measurements. By utilizing these raw voltage vectors **V** as direct inputs, the proposed framework bypasses the conventional reconstruction of the internal conductivity distribution σ.

### 2.2. Lightweight Inference Model

The core of the proposed framework is a task-oriented inference model that maps boundary voltage data directly to spatial coordinates. The localization task is formulated as a mapping function Φ with trainable parameters:

(4) $$P=\Phi \left(V;W,b\right)$$

where $V\in R^{M}$ is the input vector of normalized voltage differences obtained from the Opposite (*M* = 24) or Adjacent (*M* = 40) method. The output **P** represents the class probability distribution of the object’s location. The matrices **W** and vectors **b** denote the weights and biases, respectively, which are optimized during the training phase to minimize the categorical cross-entropy loss L:

(5) $$L=-\sum_{k=1}^{C}{\hat{P}}_{k}log\left(P_{k}\right)$$

where *C* is the number of classes (*C* = 11 for *r*-estimation and *C* = 48 for *θ*-estimation), ${\hat{P}}_{k}$ is the ground truth label, and $P_{k}$ is the predicted probability for class *k*.

This architecture is specifically designed to be lightweight by eliminating the need for iterative inverse solvers, such as Jacobian matrix updates, which are computationally prohibitive for edge devices. Instead, the localization task is reduced to a single forward-pass inference consisting of simple matrix–vector multiplications. This approach minimizes the memory footprint and ensures high-speed execution on resource-constrained hardware.

As shown in the architecture diagram (Figure 1c), the model consists of an input layer, a single fully connected hidden layer with a rectified linear unit (ReLU) activation, and an output layer with a Softmax function to yield the probability distribution. To optimize the model for edge computing environments, we varied the number of hidden layers from one to six and evaluated the trade-off between estimation accuracy and power efficiency.

In this study, two separate models were implemented to estimate *r* and *θ* independently. The *r*-estimation model produces an 11-class probability distribution $P(r)\in \;{\left[0,\;1\right]}^{11}$, corresponding to discretized levels from 1 to 41 mm. Simultaneously, the *θ*-estimation model outputs a 48-class probability distribution $P(\theta )\in \;{\left[0,\;1\right]}^{48}$ for angles ranging from 0 to 352.5° in 7.5° increments. The machine learning framework was implemented using TensorFlow 2 with Python 3.10. The implementation process for object localization using the trained model is shown in Figure 1d. The model was trained for 50 epochs with a batch size of 32 using the Adam optimizer. The learning rate was set to 0.001.

For dataset preparation, a total of 5136 samples were generated via FEM-based forward simulations, with 5084 samples used for training and 52 for testing. Each dataset consists of boundary voltage patterns corresponding to a single object characterized by Φ, *r*, and *θ*. By utilizing these dedicated models, the system can perform high-speed inference through simple matrix arithmetic, eliminating the need for iterative Jacobian updates or pixel-by-pixel reconstruction. This streamlined architecture is the primary factor enabling the significant reduction in power consumption.

### 2.3. Experimental Validation

To validate the proposed method, solution-based EIT experiments were conducted. Figure 1e shows the experimental setup and electrode configuration. A cylindrical container (100 mm in diameter) was filled with a 0.1 mol/L sodium hydroxide solution (conductivity: 2.18 S/m), and a single SUS304 cylindrical object (15 mm in diameter) was placed in the solution.

The object location was defined in polar coordinates (*r*, *θ*), and measurements were performed for selected values of *r* and *θ*. Experiments were conducted at *r* = 9, 17, 25, and 33 mm and *θ* = 0°, 7.5°, and 15°, corresponding to a subset of the simulated positions. Eight stainless steel electrodes (SUS303, 3 mm in diameter) were uniformly arranged inside the container.

A current and voltage source (155-AC, Lake Shore Cryotronics Inc., Westerville, OH, USA) was used for current injection, and voltage measurements were performed using a digital multimeter (GDM-8261A, TEXIO TECHNOLOGY CORPORATION, Yokohama, Japan). The measurements were carried out with an injection current of 5 mA and a measurement frequency of 1 kHz.

To quantitatively evaluate the discrepancy between the estimated object location probability distributions and the ideal probability distribution, the Wasserstein distance [22] was employed as an evaluation metric. A two-dimensional probability distribution of the object location, denoted as P(r,θ), was constructed as follows:

(6) $$P\left(r,\;\theta \right)=\frac{P\left(r\right)P(\theta )}{\sum_{r,\theta }P\left(r\right)P(\theta )}$$

Here, *P*(*r*) represents the probability of the object being present at the radial distance *r*, and *P*(*θ*) represents the probability of the object being present at the rotation angle *θ*. The Wasserstein distance $w_{1}(P,Q)$ is defined by the following equation [28]:

(7) $$w_{1}\left(P,\;Q\right)=\sum_{r,\theta }P\left(r,\theta \right)\left\|\left(r,\theta )-(r_{0},\theta_{0}\right)\right\|$$

Here, $\left(r_{0},\theta_{0}\right)$ denotes the object location corresponding to the ground-truth label. A smaller Wasserstein distance indicates that the estimated probability distribution is more localized around the true object position.

The computational cost and energy consumption were evaluated using a personal computer equipped with an Intel(R) Core(TM) i9-10900K CPU (3.70 GHz), an NVIDIA GeForce RTX 2080 SUPER GPU, and 16.0 GB of RAM, running Windows 11 Pro (64-bit). The proposed machine learning-based inference was implemented using Python 3.10.11, while the conventional EIDORS-based reconstruction was performed in MATLAB R2024b. The energy consumption during the execution of each process was measured using a power meter. To isolate the computational cost of the algorithms, the system’s idle-state power consumption was subtracted from the total power measured during the execution, thereby calculating the net energy consumption directly attributed to the localization tasks.

## 3. Results and Discussion

This section presents and discusses the results obtained from both numerical simulations and physical experiments to evaluate the performance of the proposed task-oriented EIT framework. The evaluation is focused on two primary aspects: localization accuracy and energy efficiency. First, we analyze the spatial existence probabilities generated by various neural networks, comparing the detection performance between the Adjacent and Opposite excitation strategies. Second, we provide a comparative analysis of computational resource requirements and power consumption against conventional inverse-problem-based reconstruction methods. Through these evaluations, we demonstrate the practical advantages of bypassing redundant image reconstruction for real-time sensing in resource-constrained environments.

### 3.1. Effect of Hidden Layer Depth on Estimation of Accuracy and Confidence

**Figure 2:**
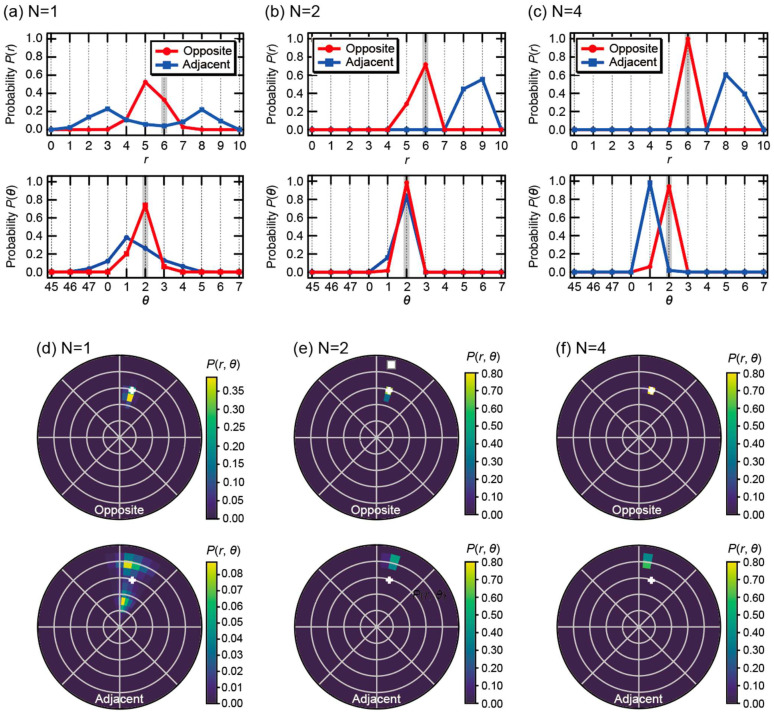
Marginal probability distributions of the object location: radial distribution *P*(*r*) and angular distribution *P*(*θ*), obtained from experimental data at (*r*, *θ*) = (6, 2) using various learning models with (**a**) *N* = 1, (**b**) *N* = 2, and (**c**) *N* = 4 hidden layers. (**d**–**f**) Schematic visualization of the spatial probability distribution *P*(*r*, *θ*) in the (*r*, *θ*) space for the corresponding models. The plus symbol indicates the ground-truth object position.

To investigate the impact of model complexity on localization performance, the number of hidden layers *N* was varied from 1 to 6. Figure 2 illustrates the evolution of the estimation results for both the Opposite and Adjacent configurations when an object was placed at (*r*, *θ*) = (6, 2) in the simulation.

When only a single hidden layer was employed (*N* = 1), significant uncertainty was observed in the estimation results for both methods, as shown in Figure 2a,d. In the Adjacent configuration, the radial probability distribution *P*(*r*) exhibited a bimodal split with peaks at *r* = 3 and *r* = 8. This phenomenon is clearly visualized in the 2D distribution in Figure 2d, where the probability mass is divided into two distinct regions flanking the true position (+). This highlights the inherent physical ambiguity of the Adjacent method, which struggles to uniquely identify objects in the central region when using a shallow network. In contrast, while the Opposite method produced a unimodal distribution at *N* = 1, the maximum probability was slightly offset toward the outer region (*r* = 5).

Increasing the depth to *N* = 2 made the performance gap between the two configurations more pronounced. As shown in Figure 2b,e, the peaks of *P*(*r*) and *P*(*θ*) for the Opposite method aligned perfectly with the ground truth, forming an extremely sharp peak at the true position (+) in the 2D plane. This suggests that the physical characteristics captured by the Opposite configuration can be extracted as highly discriminative features even by a relatively lightweight network. Conversely, the Adjacent method remained unstable, displaying a broad distribution with a wide tail far from the true position, indicating that two layers are insufficient to compensate for the lack of sensitivity in the center of the domain.

Further increasing the depth to *N* = 4 (Figure 2c,f) led to a drastic improvement in the Adjacent method, as the probability distribution finally began to converge around the true location (*r*, *θ*) = (6, 2). The Opposite method further increased its confidence, achieving pinpoint localization. Notably, increasing the number of layers to *N* = 5 and 6 did not yield significant improvements in estimation accuracy despite the increased computational load.

Table 2 summarizes the classification accuracy as a function of the number of hidden layers *N*. For the Opposite method, the radial estimation accuracy *P*(*r*) reaches a perfect score of 1.0000 at *N* = 4, with no further improvement observed at *N* = 6. Similarly, while the Adjacent method shows continued improvement, the gain in accuracy becomes marginal beyond four layers. These numerical results confirm that the model’s learning saturates at *N* = 4, which provides sufficient complexity to resolve the spatial features of the sensing domain without redundant computational overhead. Consequently, for all subsequent evaluations, we adopted the configuration with *N* = 4 hidden layers. This choice ensures a balance between high localization accuracy and low computational cost, which is essential for implementation on power-constrained edge devices.

### 3.2. Quantitative Evaluation of Localization Error

**Figure 3:**
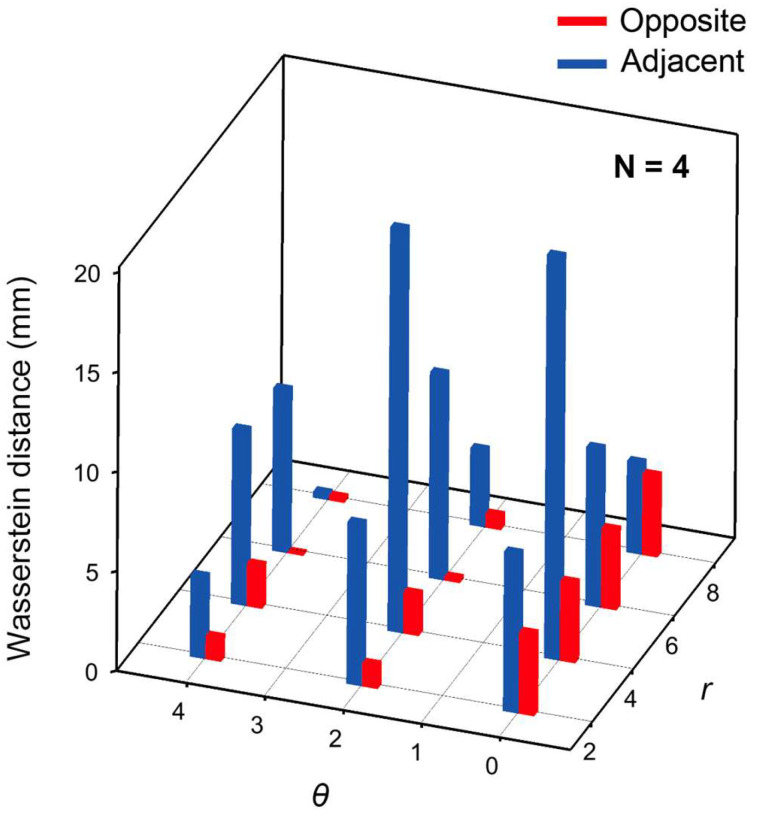
Wasserstein distance as a function of r and θ, evaluated using the optimized model with *N* = 4 hidden layers.

Figure 3 presents the Wasserstein distances (*w*_1_) of the object location probability distributions *P*(*r*, *θ*) obtained from experimental data, calculated using the optimized model with *N* = 4 hidden layers. A comparison between the Adjacent and Opposite methods shows that the Opposite method yields smaller Wasserstein distances for all object locations.

The model was fine-tuned by systematically investigating the impact of the network depth on localization performance. We evaluated architectures ranging from one to six hidden layers to identify the optimal balance between classification accuracy and computational complexity. In addition to the structural optimization, the learning rate and batch size were tuned during the training phase to ensure stable convergence of the Wasserstein-distance-based loss function. As a result, a four-layer configuration was selected as the final model, providing the highest discriminative power for both radial and angular estimations.

These results indicate that the probability distributions estimated by the Opposite method are more strongly concentrated around the ground-truth labels than those obtained by the Adjacent method. In other words, the Opposite method exhibits lower ambiguity in object localization and enables more unique and reliable position estimation.

From these results, it is evident that differences in current injection and voltage measurement configurations have a significant impact on object localization performance. The higher positional discriminability observed with the Opposite method is attributed to the fact that the current injection axis traverses the entire domain, thereby enhancing both the angular dependence of the voltage distribution and the sensitivity to deeper regions of the medium. As a result, the amount of informative content contained in the boundary voltage differences increases, and features related to the object location become more pronounced.

In contrast, in the Adjacent method, the injected current predominantly flows in the vicinity of the electrodes, leading to a relatively localized sensitivity distribution within the domain. This reduces discriminability in both the angular and radial directions. Consequently, the probability distributions estimated by the machine learning model become more spread out, resulting in increased uncertainty in object localization.

Notably, although the Adjacent method has a higher input dimensionality (40 dimensions) than the Opposite method, it does not necessarily achieve superior estimation accuracy. This result suggests that object localization performance is governed not by the number of input features itself, but by the structure of the physical sensitivity distribution inherent in the current injection and measurement configuration. Specifically, the global current traversal in the Opposite method results in a more uniform sensitivity matrix, where the spatial gradients of the boundary voltages are more linearly independent across the entire domain. This physical property reduces the ‘dead zones’ in the feature space, allowing the neural network to map input voltage patterns to spatial coordinates with higher class separability, even for objects located deep within the medium.

In addition, the two-dimensional probability distributions shown in Figure 2b do not represent reconstructed conductivity images, but rather directly visualize the existence probability of the object center. This representation demonstrates that the positional information embedded in EIT measurement data can be quantitatively and intuitively evaluated without solving the inverse problem.

Furthermore, while the current evaluation is based on a fixed object size (15 mm), the proposed framework’s performance is expected to be influenced by variations in object size and conductivity. Larger objects generally enhance the signal-to-noise ratio of boundary voltages, potentially improving localization accuracy, though they may introduce ambiguity regarding the object’s precise center of mass. Conversely, smaller or low-contrast objects would present challenges due to subtler voltage perturbations. Addressing these variations through multi-scale training datasets remains a subject for future investigation to further validate the general applicability of this task-oriented approach.

### 3.3. Computational Cost and Energy Consumption

To evaluate the computational cost of object localization, energy consumption on a PC was compared between a conventional inverse-problem-based reconstruction method using EIDORS and the proposed lightweight machine learning approach under the same computational environment. The EIDORS-based method consumed 3.09 Wh, whereas the proposed approach (*N* = 1) consumed 0.96 Wh for the Opposite method and 0.94 Wh for the Adjacent method.

While these measurements were performed with a single hidden layer, the increase in computational load for the optimized *N* = 4 model is negligible compared to the overall system overhead and the heavy iterative processing required by conventional inverse solvers. In fact, the inference time remained nearly constant regardless of the layer depth within this range. These results confirm that the proposed framework achieves a substantial reduction in energy consumption (approximately 70%) while maintaining high localization accuracy. This efficiency stems from the direct estimation of coordinates from low-dimensional features, bypassing the need for high-dimensional image reconstruction.

It should be noted, however, that while these results demonstrate significant energy savings under ideal simulation conditions, the sensitivity to measurement noise (e.g., SNR < 40 dB) remains a critical factor for clinical deployment. Future work will incorporate noise-robust training to further validate the system’s reliability in real-world sensing environments.

## 4. Conclusions

In this study, we proposed a lightweight machine learning approach for directly estimating object center positions from EIT boundary voltage measurements, bypassing the need for computationally expensive inverse-problem-based image reconstruction. By employing FEM-generated datasets and experimental validation, the object localization performance was systematically compared between the Opposite and Adjacent current injection configurations.

The results demonstrated that the Opposite method yields significantly more localized probability distributions and smaller Wasserstein distances compared to the Adjacent method. Specifically, the optimized model with four hidden layers (*N* = 4) achieved a perfect classification accuracy (1.00) for radial estimation in the Opposite configuration, whereas the Adjacent method exhibited inherent ambiguity due to its localized sensitivity distribution. These findings highlight that the localization performance is primarily governed by the physical current field structure rather than the dimensionality of the input features.

Furthermore, experimental measurements confirmed that the proposed approach reduces energy consumption by approximately 70% compared to conventional EIDORS-based reconstruction. This efficiency stems from the task-oriented inference framework, which directly maps boundary voltages to the spatial coordinates of interest. While this method does not aim to reconstruct detailed conductivity distributions, it provides sufficient localization accuracy and robustness for practical applications while drastically reducing computational overhead.

The framework presented in this study is ideally suited for EIT applications in resource- and power-constrained environments, such as mobile sensing and embedded systems. This approach is expected to facilitate the development of next-generation, low-power EIT-based sensor systems for real-time object tracking and industrial monitoring.

## Figures and Tables

**Table 1:** Comparison of representative EIT methods and the proposed framework.

Reference	Primary Output	Inverse Problem	Computational Cost
Adler et al. [1]	Full conductivity map (iterative image recon.)	Yes	3.09 Wh (measured in this study)
Minakawa et al. [9]	High-resolution conductivity map	Yes	Not reported
Wanta et al. [10]	Conductivity map (real-time)	Yes	Not reported
Zou et al. [23]	3-D tumor boundary map	Yes	Not reported
Zhang et al. [24]	Conductivity distribution image	Yes	Not reported
Li et al. [25]	Boundary impedance data (recon. done separately)	Yes	1.76 mW (hardware only)
Toivanen et al. [26]	3-D stroke monitoring image (diff. conductivity)	Yes	Not reported
Shi et al. [27]	Conductivity map (state-of-the-art quality)	Yes	Not reported
Proposed (this study)	Position probability	Not required	0.96 Wh

**Table 2:** Probabilities using various learning models.

Mode	Probability	Number of Hidden Layer *N*					
		1	2	3	4	5	6
Opposite	*P*(*r*)	0.79	0.92	0.94	1.00	1.00	0.98
	*P*(*θ*)	0.92	0.94	0.96	0.92	0.94	0.94
Adjacent	*P*(*r*)	0.50	0.58	0.62	0.67	0.62	0.73
	*P*(*θ*)	0.44	0.88	0.90	0.92	0.85	0.92

## Data Availability

The data and code supporting the findings of this study are available in the 2026/TI_sensors directory of the GitHub repository https://github.com/TUS-ikunolab/public_data (accessed on 17 April 2026).
